# Supplementary material for: Transmission of SARS-CoV-2 from humans to animals and potential host adaptation
Source: Nat Commun. 2022 May 27;13:2988. doi: 10.1038/s41467-022-30698-6 (PMC9142586; doi:10.1038/s41467-022-30698-6)
Supplement: Supplementary file 1 — Supplementary Information [file 41467_2022_30698_MOESM1_ESM.pdf]

Supplementary Information:

Transmission of SARS-CoV-2 from humans to animals and potential host adaptation

Tan et al.

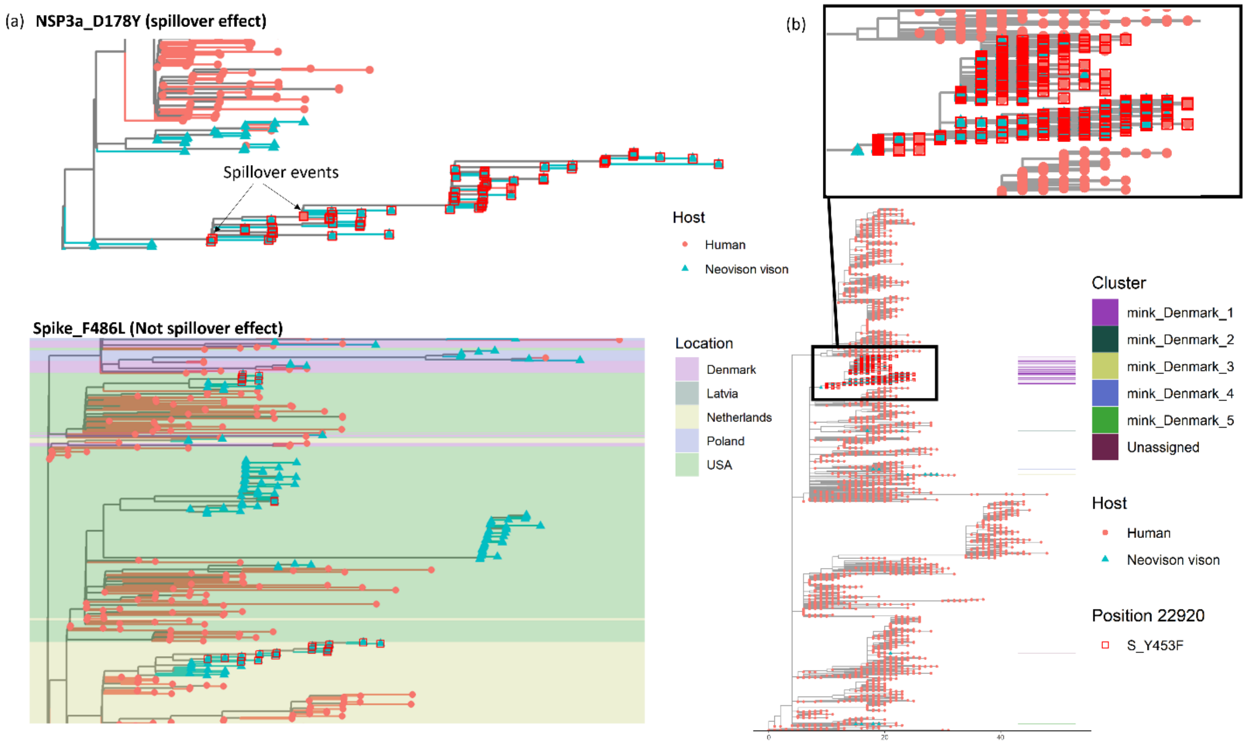

Figure S1. Spillover effect and its lack thereof. (a) Subtrees of mink-human phylogenies illustrating the cases where a mutation is already present in the ancestral human lineage prior to spillover (NSP3a_D178Y) and where the mutation arose in animals following spillover (Spike_F486L). (b) Danish mink-human phylogeny comprising isolates collected before 1 December 2020 (n = 15,895) and a magnified view of the mink_Denmark_1 cluster. Tree tips and terminal branches are coloured by host of isolation. Tips where the respective mutations are present are indicated by red squares.

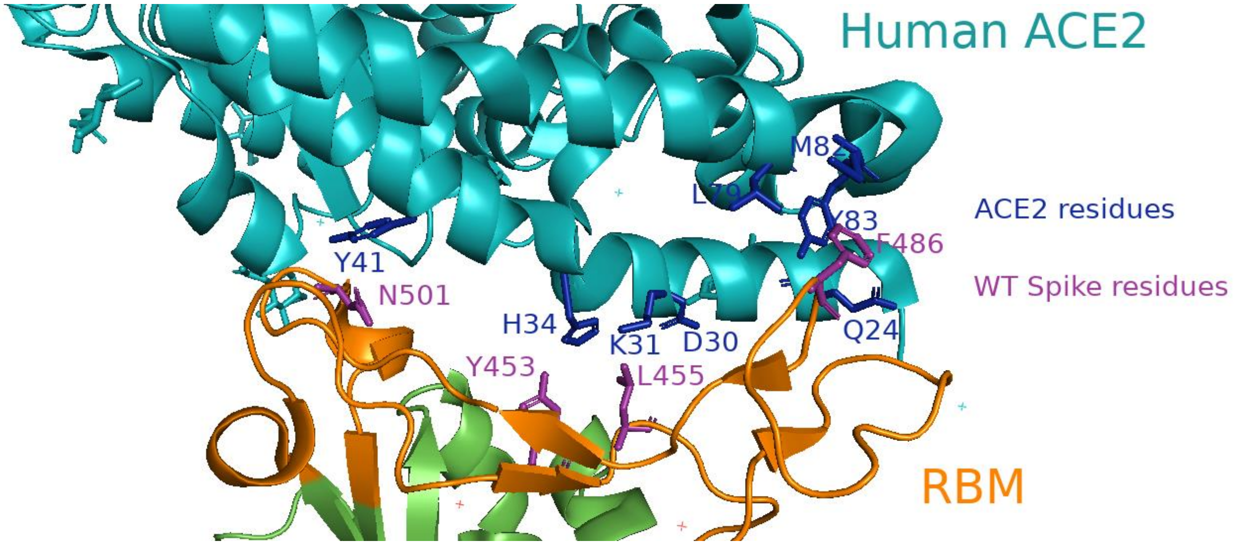

***Figure S2.* Protein structure of the Spike receptor binding motif (RBM) of the receptor binding domain (RBD) in complex with human ACE2**. Human ACE2, the RBM and the rest of RBD are shown in cyan, orange and green respectively. The wild-type amino acid residues for the candidate mutations Y453F, F486L, N501T are shown in purple. ACE2 residues reported previously to be interacting with the RBM residues L455 (in close proximity to F453), F486 and N501 are shown in blue. The Protein Data Bank (PDB) code for the SARS-CoV-2 RBM–ACE2 complex is 6M0J. This figure was rendered using *PyMOL v2.4.1*.
