# Supplementary material for: Transmission of SARS-CoV-2 from humans to animals and potential host adaptation
Source: Nat Commun. 2022 May 27;13:2988. doi: 10.1038/s41467-022-30698-6 (PMC9142586; doi:10.1038/s41467-022-30698-6)
Supplement: Supplementary file 3 — Description of Additional Supplementary Files [file 41467_2022_30698_MOESM3_ESM.pdf]

Description of Additional Supplementary Files

Tan et al.

File Name: Supplementary Data 1

Description: Metadata of 20 putative mink- and 34 deer-specific candidate mutations.

File Name: Supplementary Data 2

Description: Metadata of 16,911 isolates representing the global context of animal-associated SARS-CoV-2 infections.

File Name: Supplementary Data 3

Description: Metadata of 1,189,519 human and mink isolates used for manually identifying phylogenetically distinct clusters.

File Name: Supplementary Data 4

Description: Metadata of 1,698,656 human and deer isolates used for manually identifying phylogenetically distinct clusters.

File Name: Supplementary Data 5

Description: Reconstructed consensus phylogeny of 928 mink SARS-CoV-2 isolates, rooted to WIV04, and using UFBoot and SH-aLRT for 1000 replicates. This phylogeny is annotated with UFBoot and SH-aLRT support scores in blue and red respectively.

File Name: Supplementary Data 6

Description: Reconstructed consensus phylogenies of 95 deer SARS-CoV-2 isolates, rooted to WIV04, and using UFBoot and SH-aLRT for 1000 replicates. This phylogeny is annotated with UFBoot and SH-aLRT support scores in blue and red respectively.

File Name: Supplementary Data 7

Description: Cluster metadata for animal isolates. These clusters were manually identified and assigned names with the format ‘*host-country-cluster number*’.

File Name: Supplementary Data 8

Description: Metadata of 1,769 mink and human background 1 isolates used for the allele frequency and homoplasy analyses.

File Name: Supplementary Data 9

Description: Metadata of 22,381 mink and human background 2 used for the allele frequency and homoplasy analyses.

File Name: Supplementary Data 10

Description: Metadata of 189 deer and human background 1 used for the allele frequency and homoplasy analyses.

File Name: Supplementary Data 11

Description: Metadata of 7,325 deer and human background 2 sequences used for the allele frequency and homoplasy analyses.

File Name: Supplementary Data 12

Description: The metadata of the final 1,327 mink and human background 1 isolates used for the substitution rate analyses.

File Name: Supplementary Data 13

Description: The metadata of the final 187 deer and human background 1 deduplicated sequences with unambiguous sampling dates used for the substitution rate analyses.
