# Supplementary figures and images for: Transmission of SARS-CoV-2 from humans to animals and potential host adaptation
Source: Nat Commun. 2022 May 27;13:2988. doi: 10.1038/s41467-022-30698-6 (PMC9142586; doi:10.1038/s41467-022-30698-6)

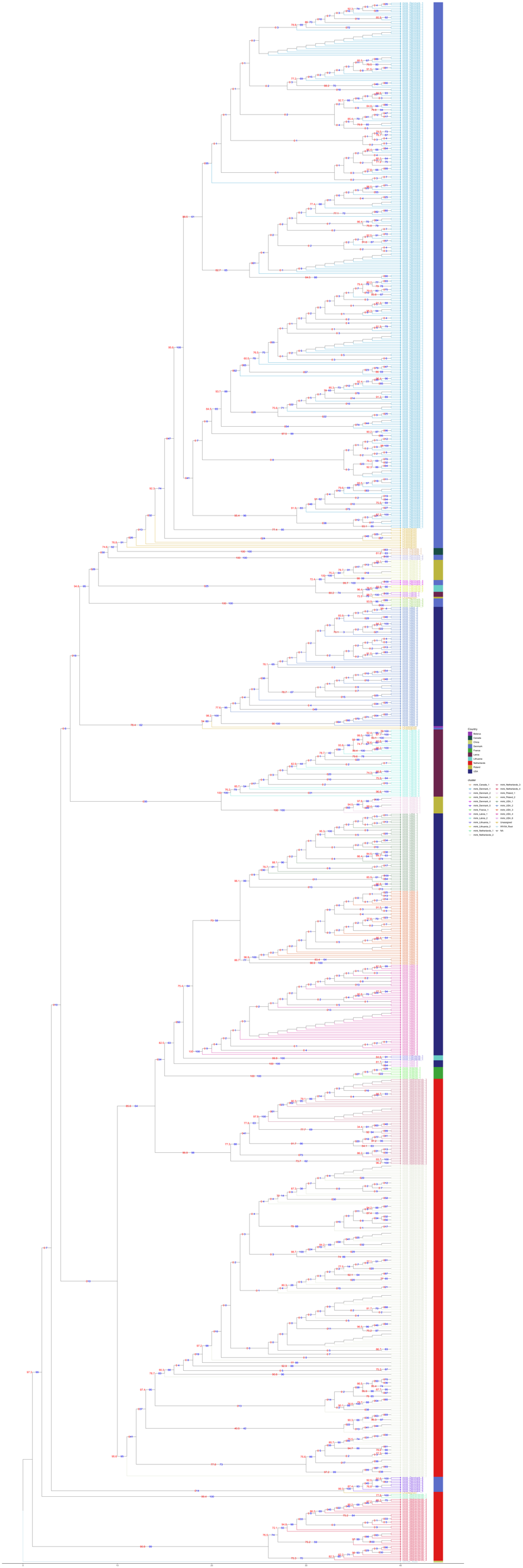

Supplement: Supplementary file 4 — Supplementary Data 1-13 [file 41467_2022_30698_MOESM4_ESM.zip › supplementary_data/Supplementary_Data_5_all_mink_bootstrap_tree.sh-aLRT.parsed.pdf]

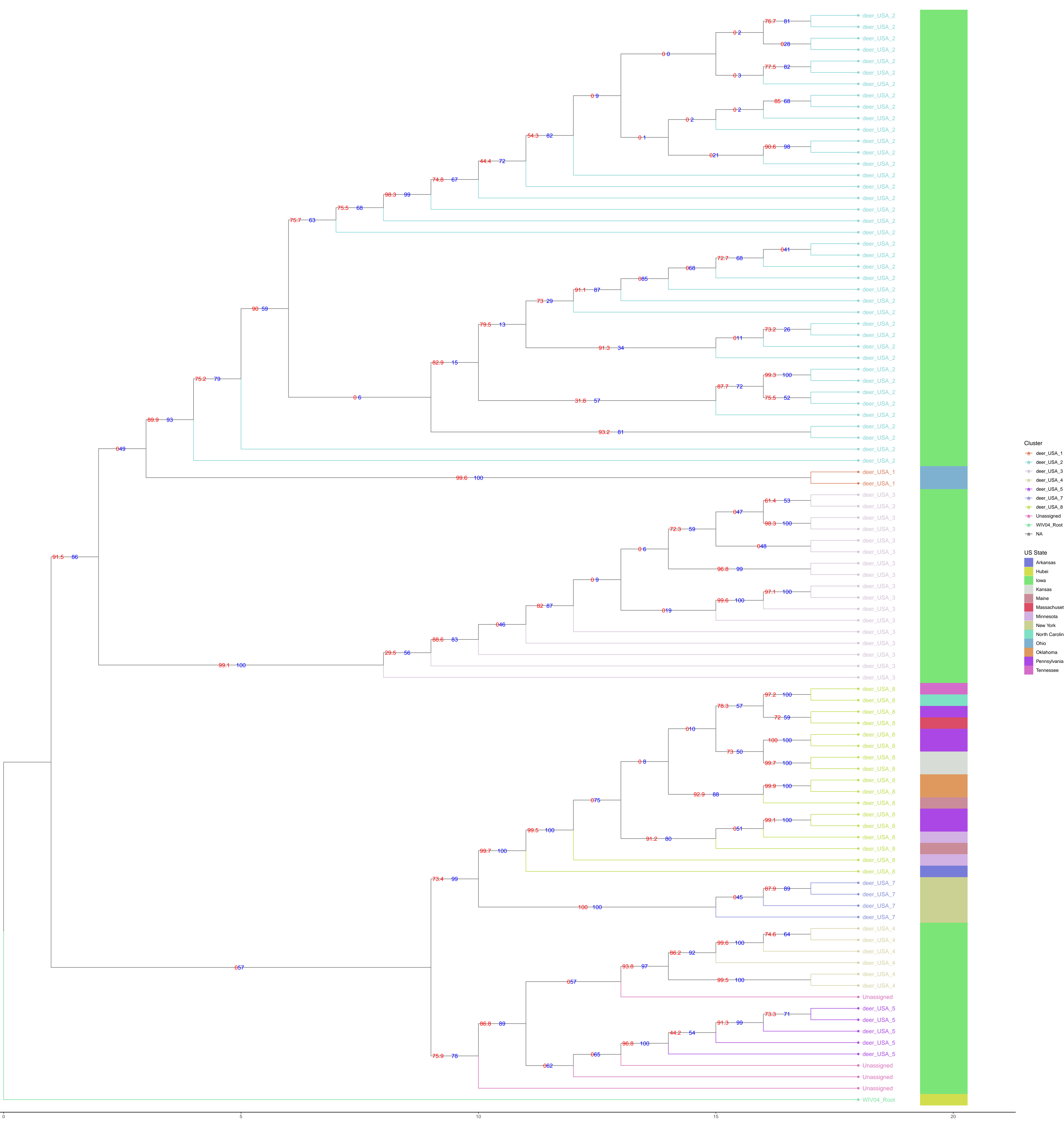

Supplement: Supplementary file 4 — Supplementary Data 1-13 [file 41467_2022_30698_MOESM4_ESM.zip › supplementary_data/Supplementary_Data_6_all_deer_bootstrap_tree.sh-aLRT.parsed.pdf]
