# Supplementary material for: Transmission of SARS-CoV-2 from humans to animals and potential host adaptation
Source: Nat Commun. 2022 May 27;13:2988. doi: 10.1038/s41467-022-30698-6 (PMC9142586; doi:10.1038/s41467-022-30698-6)
Supplement: Supplementary file 5 — Reporting Summary [file 41467_2022_30698_MOESM5_ESM.pdf]

nature portfolio

Corresponding author(s): Cedric C.S. Tan

Last updated by author(s): May 8, 2022

Reporting Summary

Nature Portfolio wishes to improve the reproducibility of the work that we publish. This form provides structure for consistency and transparency in reporting. For further information on Nature Portfolio policies, see our Editorial Policies and the Editorial Policy Checklist.

Statistics

For all statistical analyses, confirm that the following items are present in the figure legend, table legend, main text, or Methods section.

n/a	Confirmed	
☐	☒	The exact sample size (*n*) for each experimental group/condition, given as a discrete number and unit of measurement
☒	☐	A statement on whether measurements were taken from distinct samples or whether the same sample was measured repeatedly
☐	☒	The statistical test(s) used AND whether they are one- or two-sided<br>*Only common tests should be described solely by name; describe more complex techniques in the Methods section.*
☒	☐	A description of all covariates tested
☒	☐	A description of any assumptions or corrections, such as tests of normality and adjustment for multiple comparisons
☐	☒	A full description of the statistical parameters including central tendency (e.g. means) or other basic estimates (e.g. regression coefficient) AND variation (e.g. standard deviation) or associated estimates of uncertainty (e.g. confidence intervals)
☐	☒	For null hypothesis testing, the test statistic (e.g. *F*, *t*, *r*) with confidence intervals, effect sizes, degrees of freedom and *P* value noted<br>*Give P values as exact values whenever suitable.*
☐	☒	For Bayesian analysis, information on the choice of priors and Markov chain Monte Carlo settings
☒	☐	For hierarchical and complex designs, identification of the appropriate level for tests and full reporting of outcomes
☒	☐	Estimates of effect sizes (e.g. Cohen's *d*, Pearson's *r*), indicating how they were calculated

Our web collection on statistics for biologists contains articles on many of the points above.

Software and code

Policy information about availability of computer code

Data collection	No software was used for data collection.

Data analysis	R 4.1.2<br>Python 3.7.11<br>Seqtk 1.3<br>IQ-Tree2 2.1.4-beta COVID-edition<br>Dendroscope 3<br>Ape v5.5<br>HomoplasyFinder v0.0.0.9<br>ggtree v3.2.1<br>tidyverse v1.3.1<br>data.table v1.14.2<br>seqkit v2.2.0<br>TreeTime v.8.5<br>PROVEAN web server<br>PyMOL v2.4.1<br>MODELLER v.9.24<br>HADDOCK v2.4<br>mCSM-PPI2 server<br><br>https://github.com/cednotsed/ditto.git<br>https://github.com/cednotsed/SARS-CoV-2-hookup

For manuscripts utilizing custom algorithms or software that are central to the research but not yet described in published literature, software must be made available to editors and reviewers. We strongly encourage code deposition in a community repository (e.g. GitHub). See the Nature Portfolio guidelines for submitting code & software for further information.

Data

Policy information about availability of data

All manuscripts must include a data availability statement. This statement should provide the following information, where applicable:

- Accession codes, unique identifiers, or web links for publicly available datasets
- A description of any restrictions on data availability
- For clinical datasets or third party data, please ensure that the statement adheres to our policy

All genomic sequences used in this study are publicly available on registration at GISAID (https://www.gisaid.org/). The accessions for all sequences analysed are listed in Supplementary Data 2-4, 8-13. All source data required to reproduce the analyses in this paper can be downloaded from Zenodo (https://doi.org/10.5281/zenodo.6528187). The structure of the wild-type SARS-CoV-2-Spike:human-ACE2 can be downloaded from the PDB under the accession 6M0J (https://www.rcsb.org/structure/6m0j).

Field-specific reporting

Please select the one below that is the best fit for your research. If you are not sure, read the appropriate sections before making your selection.

☒ Life sciences ☐ Behavioural & social sciences ☐ Ecological, evolutionary & environmental sciences

For a reference copy of the document with all sections, see nature.com/documents/nr-reporting-summary-flat.pdf

Life sciences study design

All studies must disclose on these points even when the disclosure is negative.

Sample size	We used all high quality animal-associated genomes hosted on GISAID. We limited our analyses to sample groups where n > 50, which is sufficiently large for all our statistical analyses used.
Data exclusions	We excluded human SARS-CoV-2 isolates that were documented as arising from animal-to-human spillback events. These samples would confound analysis of host-specific genomic changes.
Replication	The samples used in this study are a result of intensive global sequencing efforts. It is quite unlikely that a dataset of this sort can be reproduced.
Randomization	Not relevant as we analysed publicly available data. SARS-CoV-2 sequences were randomly sampled according to constraints described in the methods.
Blinding	Not relevant as we analysed publicly available data.

Reporting for specific materials, systems and methods

We require information from authors about some types of materials, experimental systems and methods used in many studies. Here, indicate whether each material, system or method listed is relevant to your study. If you are not sure if a list item applies to your research, read the appropriate section before selecting a response.

Materials & experimental systems

n/a	Involved in the study
☒	☐ Antibodies
☒	☐ Eukaryotic cell lines
☒	☐ Palaeontology and archaeology
☒	☐ Animals and other organisms
☒	☐ Human research participants
☒	☐ Clinical data
☒	☐ Dual use research of concern

Methods

n/a	Involved in the study
☒	☐ ChIP-seq
☒	☐ Flow cytometry
☒	☐ MRI-based neuroimaging
